# Association of Glutathione-S-Transferases M1 and T1 Deletional Variants with Development of Oral Squamous Cell Carcinoma: A Study in the South-East of Iran

**DOI:** 10.31557/APJCP.2019.20.6.1921

**Published:** 2019

**Authors:** Shirin Saravani, Masoud Miri-Moghaddam, Ali Bazi, Ebrahim Miri-Moghaddam

**Affiliations:** 1 *Oral and Dental Disease Research Center, Department of Oral and Maxillofacial Pathology, Faculty of Dentistry, *; 2 *Students Scientific Research Center, School of Dentistry, *; 5 *Genetics of Non-Communicable Disease Research Center, Zahedan University of Medical Sciences, Zahedan, *; 3 *Clinical Research Development Unit, Amir-Al-Momenin Hospital, Zabol University of Medical Sciences, Zabol, *; 4 *Cardiovascular Diseases Research Center and Department of Molecular Medicine, Faculty of Medicine, Birjand University of Medical Sciences, Birjand, Iran. *

**Keywords:** Squamous cell carcinoma, glutathione S, transferase T1, glutathione S, transferase M1

## Abstract

**Background:**

The role of genetic polymorphisms in genes of Glutathione-S-transferases (GST) enzymes in susceptibility to oral cavity cancers is controversial. Oral Squamous Cell Carcinoma (OSCC) is the most common oral cavity neoplasm. Aimed to evaluate the potential impacts of two well-known null variants residing in the gene encoding GSTM1 and GSTT1 enzymes of OSCC patients in the southeast of Iran.

**Methods:**

In a case-control design, 113 individuals (50 OSCC patients, and 63 healthy subjects) were included. DNA was extracted using paraffin-embedded tissues. GST genotyping was carried out using multiplex PCR.

**Results:**

In 113 participants, 41 (36.3%) and 72 (63.7%) were males and females respectively. No significant difference was recognized for distribution of GSTM1 (P=0.11) and GSTT1 (P=0.28) null genotypes between OSCC patients (58%, and 24% respectively) and healthy controls (42.9% and 15.9% respectively). Also, no significant difference was noted regarding the frequency of GSTM1 null genotype in different histological grades, however, those patients with more aggressive disease (poorly differentiated or grade III) revealed with a significantly higher ratio (66.7%) of GSTT1 null genotype (P=0.002). The highest odds ratio for OSCC was related to combined null genotypes for GSTM1 and GSTT1 (OR=2.5, 95% CI: 0.7-9.2), however, this was not statistically significant finding (P=0.15).

**Conclusion:**

Null genotypes polymorphisms were more common in OSCC than healthy individuals. GSTT1 null genotype may be an important genetic factor in the progression of OSCC.

## Introduction

Oral squamous cell carcinoma (OSCC) is a subcategory of carcinomas involving the head and neck. OSCC constitutes nearly 90% of cancers residing in the oral cavity. OSCC has a poor outcome and about half of the patients with this cancer experience 5 years survival (Leemans et al., 2011). Tobacco and alcohol consumption have been proposed as the major risk factors for SCC (Olivieri et al. 2009). Although the importance of genetic factor in the development of oral cancer is accepted, its exact molecular mechanism in carcinogenesis process is not specified yet (Dong et al., 2013).

Glutathione-S-transferases (GST) are detoxifying enzymes participating in the neutralization of oxidative mediators within cells. GSTs belong to phase II detoxifying enzymes mediating conjugation of carbon, nitrogen or sulfur-containing molecules. In mammals, eight families of GSTs have been identified. Based on the similarity of the gene sequences and type of substrate, these are divided into Alpha (GTS A), Mu (GTS M), Teta (GTS T), Pi (GTS P), Zeta (GTS Z), Sigma (GTS S), Kappa (GTS K), and Omega (GTS O) categories. Of these, polymorphisms of GTST1 and GTSM1 genes have been studied more than others. GTSM1 and GSTT1 genes have been mapped at 1p13.3 and 22q11.2 chromosomal locations, respectively. Indeed, homozygote deletions have been frequently reported in these loci, result in non-functional protein product or enzyme activity (Nakajima et al., 1996). 

GTST1 and M1 are enzymes that are involved in detoxification of various carcinogenic and oxidative mediators. GSTT1 is generally responsible for scavenging of carcinogens available in tobacco smoke (such as mono haloethanes and ethylene oxide), while GSTM1 isozymes are scavengers of polyaromatic hydrocarbons (PAH) and benzopyrene (Nakajima et al., 1996). Nevertheless, the activity of these enzymes generally overlaps with each other.

Role of GST enzymes in OSCC is controversial. The expression of GST isoforms has been suggested as a diagnostic biomarker and a disease-monitoring factor in cancers of squamous cells (Li et al., 2014). Zakiullah et al., (2015) indicated that non-functional GSTT1 and GSTM1 genes led to increasing oral cancer by three-times. Furthermore, they showed that simultaneous deletion of one allele from each gene of these variables significantly increased the chance for cancer incidence. While Buch et al., (2008) could find no significant relationship between the genetic variation of these genes and metabolic enzymes in oral and oropharyngeal cancers. 

Regarding the role of GTS enzymes in detoxification of various carcinogens, it has been suggested that genetic polymorphisms of GTS enzymes may be related to cancer (Benhamou et al., 2002; Singh et al., 2008). Genes of GTSM1 and GTST1 show deletional alleles which homozygous state resulting in no enzyme activities. Polymorphisms in these genes can increase the susceptibility of individuals to toxic and oxidative agents. The role of deletional polymorphisms of GTSM1 and GTST1 in oral cavity cancers have been studied in different geographic populations and showed different results. Nevertheless, there has been no study on the role of these polymorphisms on OSCC susceptibility in the south-east of Iran. In the present study, we aimed to address potential impacts of these genetic determinants in risk of OSCC in an Iranian population.

## Materials and Methods

*Patients and Methods*

Fifty patients diagnosed with OSCC were included. In parallel, 63 healthy subjects with no history of neoplastic conditions were served as controls. The patients and controls were sex, ethnic and age-matched. The study was conducted in 2016 in the Faculty of Dentistry of Zahedan University of Medical Sciences. Informed consent was taken from case and control groups and the study was approved by ethical committee of Zahedan University of Medical Sciences (IR.ZAUMS.REC.1395. 153).

*Diagnosis of OSCC*

The diagnosis was confirmed by two independent pathologists. The samples were accordingly categorized based on the histopathological features into three grades included well-differentiated, moderately differentiated, and poorly differentiated (grade I, II, III respectively) (Neville et al., 2015).

*DNA isolation and confirmation*

The DNA samples extracted from paraffin-embedded tissue in the case group, while blood samples were used for the control group. Briefly; the paraffin removed by immersing the samples into Xylene, ethanol 100%, ethanol 80%, ethanol 50% and then overnight incubation in 1 ml distilled water at 4°C. Protein digestion was carried out using nucleic acid lysis buffer [(10 mM Tris Base (1.21 g/L), 400 mM NaCl (32.4 g/L), 2 mM Na2EDTA (0.75 g/L), 0.7% SDS (7.0 g/L)] and proteinase K enzyme. DNA was precipitated using NaCl 6M and ethanol 100%. Finally, DNA was solved in Tris-EDTA buffer.

*Genotyping*

A single assay using a multiplex PCR was performed for GSTM1 and GSTT1 genotyping while CYP1A1 gene served as control. DNA was amplified with GSTM1 forward (5’ GAA CTC CCT GAA AAG CTA AAG C 3’) and reverse (5’ GTT GGG CTC AAA TAT ACG GTG G3’) primers, and GSTT1 with forward (5’ TTC CTT ACT GGT CCT CAC ATC TC 3’) and reverse (5’TCA CCG GAT CAT GGC CAG CA 3’) primers. As an internal control, exon 7 of the CYP1A1 gene was also amplified, using forward (5’ GAA CTG CCA CTT CAG CTG TCT 3’) and reverse (5’ CAG CTG CAT TTG GAA GTG CTC 3’) primers (Abdel-Rahman et al. 1996). Amplification program included an initial denaturation (94^o^C for 5 min) followed by 30 cycles of denaturation (95 for 1 min), annealing (60 for 30 s), and extension (72 for 30 s) and a final extension (72ºC for 5 min). PCR products from co-amplification of GSTM1 (215 bp) and GSTT1 (480 bp) were visualized on ethidium bromide-stained 2.0% agarose gel. Presence of the particular allele was designated as wild genotype (positive) and a homozygous absence or deletion of the allele was designated as null genotype.

*Statistical analysis*

Statistical methods were performed in SPSS 19 software (SPSS Inc., Chicago, IL). Descriptive statistics were used for presenting frequencies of different genotypes within the patients and controls. Univariate association of the polymorphisms with case and control groups and different histopathological grades were assessed by chi-square test. Finally, logistic regression was conducted to find if any of selected variables could predict the development of OSCC. A statistical significant level was considered as p<0.05.

## Results

From overall 113 individuals, 41 (36.3%) and 72 (63.7%) were males and females respectively. There was no significant difference in male and female distribution comparing healthy controls [24(38.1%) and 39(61.9%) respectively] and OSCC patients [17(34%) and 33(66%) respectively]. The mean age of the patients and controls were 59.3±13.1 and 50.7±11.5 years old. Table 1 shows clinical features of the OSCC patients. 

No significant difference was recognized in the distribution of GSTM1 and GSTT1 genotypes between OSCC patients and healthy controls (Table 2). However, significant associations were detected between GSTT1 genotype and OSCC grade, as a majority of patients in grade I and II expressed the wild genotype, while a higher ratio of patients was identified with null GSTT1 allele among those with grade III disease (P=0.002, Table 3). Although logistic regression revealed a higher risk of OSCC for null carriers of GSTM1 (-) and GSTT1 (-) genotypes (OR=1.8 and 1.7 respectively), this was not statistically significant (Table 4).

## Discussion

Regarding the distribution of GTSM1 and GSTT1 deleted variants, the respective frequencies were 58% and 24% in OSCC patients. These frequencies were not significantly different from the ratios observed in healthy matched controls (43% and 16% respectively). Furthermore, 14% of our patients were found with homozygous deletions of GTSM1 and GTST1, while the frequency of this combination was 8% in the controls. The prevalence of these variants in different populations was between 54% to 87% for null-GSTM1 (Koch et al., 2010; Tanwar et al., 2015) and 17.6% to 47.5% for null-GSTT1 (Zakiullah et al., 2015; Peters et al., 2006). We found no Iranian studies reporting these genotypes in OSCC patients. However, in Iranian patients with esophageal squamous cell carcinoma, 43.9% and 24.3% showed null genotypes of M1 and T1 respectively which was not significantly different from healthy subjects (Moaven et al., 2010). From 28 patients with OSCC in Germany, 54% showed null genotype in GTSM1 (Koch et al., 2010). In another study in Germania OSCC cases, frequencies of M1 and T1 null genotypes were 57% and 22% respectively and have been showed no significant difference compared with healthy subjects (Kruger et al., 2015). In Brazilian OSCC patients, M1 null genotype was reported in 70% and 48% in the patients and controls (Drummond et al., 2004). The results in OSCC Indonesian, patients showed that no statistically significant differences regarding penetrance of GSTM1 and GSTT1 null genotypes (60.5% and 45.7% respectively) compared to controls (55.6% and 41.4% respectively) (Amtha et al., 2009). In a study in the United States, frequencies of both M1 and T1 null genotypes were significantly higher in patients with SCC of head and neck in comparison with control individuals (53% vs 42% for M1, and 32% vs 17% for T1 respectively); while frequencies of null genotype for both loci were 20% in cases and 8% in controls (Cheng et al., 1999). The frequencies of M1 and T1 null genotypes reported in the present study are similar to the ratios previously stated in oral cancer patients. Distribution of these genotypes seems to be variable among different geographical and ethnical strict. 

Overall, both null genotypes of GTSM1 and GTST1 rendered higher risk for OSCC in our study (ORs of 1.8 and 1.7 for M1 and T1 null genotypes respectively). Nevertheless, the higher risks were not statistically significant. The risk was considerably elevated (OR=2.5) in the state of combined M1 and T1 null genotypes, however, still, this did not reach the statistically significant level. In parallel, the presence of both M1 and T1 null genotypes elevated the risk of OSCC in Germans (Gronau et al., 2003). Null genotypes of M1 and T1 polymorphisms have been noted as independent risk factors for squamous cell carcinoma of head and neck (Cheng et al., 1999). Association of M1 null genotype with cancer was not supported by a meta-analysis of 19 case-control studies on different cancers (Peng et al., 2013). Reduced activity of GTS system can promote the higher concentration of deleterious agents that predispose to genetic instability and mutations. Reactive carcinogenic agents are neutralized by the act of these enzymes through conjugation with glutathione, a reaction catalyzed by GSTs. This can be considered as an explanation for higher risk of carcinogenesis in individuals with null genotypes of M1 and T1 polymorphisms. On the other hand, GST enzymes can promote dual roles in cancer pathogenesis by acting both as detoxifying enzymes, as well as cellular regulators in response to various stimuli (Ma et al., 2015). Lower thresholds of enzymatic activities of GSTs may benefit cancerous cells by providing a more tolerable microenvironment. Interactions of some unknown factors modulating the role of GST enzymes are yet to be elucidated. 

Interaction of genetic and environmental conditions to the risk of SCC has been demonstrated. Sharma et. al further demonstrated that smoking significantly increased the risk of SCC in GSTT1 null cases (OR=6.33; 95% CI=1.0-44.1), however, such effects were not noted for GSTM1 null individuals (Sharma et al., 2006). The odds ratio of SCC associated with the GSTM1 null genotype was 5.7 (95% CI 2.0-16.3) in cigarette smokers, 3.7 (95% CI 2.0-7.1) in tobacco chewers, 3.7 (5% CI 1.3-7.9) in bidi smokers (Buch, Notani, and Bhisey 2002). One of the limitations of our study was the unavailability of a history of smoking in our participants. Overall, a gene-environmental relationship may be responsible for differences observed for various susceptibilities to cancer in carriers of either M1 or T1 null genotypes.

Genetic loci of M1 and T1 may interact with other genetic loci involving in detoxification pathways. Relationship of cytochrome P450 (CYPs) and GTSs has been proposed. Combination of null genotypes of M1 and T1 genes with polymorphism of T6235C in CYP1A1 markedly elevated the risk of HNSCC in Brazilian patients (Lourenco et al., 2011). GTS enzymes metabolize reactive substances derived from the action of CYPS on various carcinogens. The synergy between the two systems is comprehensible as a function of these enzymatic pathways is complementary to each other, with GTS enzymes accomplishing the detoxification pathway initiated by CYPs (Olivieri et al., 2009). M1 null allele may also contribute to the risk of SCC in combination with polymorphism of two other detoxicating genes; NAT2 and XPD (Gajecka et al., 2005). M1 and T1 genetic loci interact with each other, and their interaction with other genetic loci are yet to be clarified. Because the redundant function of thee enzymes, it seems that compensatory activities may be responsible for different results reported by researchers.

Ethnicity may be important factors in determining susceptibility to oral cancer for M1 and T1 alleles (Zhao et al., 2014; Liu et al., 2015). In a meta-analysis, M1 null genotype was found as risk factor for oral neoplasm in Asian but not Caucasians (Zhao et al., 2014). In another meta-analysis, GSTT1 null genotype was identified as a risk factor for cancer of oral cavity in Asians (Dong et al., 2013). Another report demonstrated that Asians with null T1 and M1 genotypes are more likely to develop head and neck cancers than individuals with similar genotypes of European or American origin (Masood et al., 2013). The role of ethnicity should be more established by performing studies in different populations across the world. 

We detected no significant difference in penetrance of GTSM1 null genotypes regarding SCC grade, however, the majority (66.7%) of patients with grade III disease showed null genotype for GTST1 (P=0.002). On the other hand, 76.2% and 95% of the patients with disease of grade I and II were identified with positive T1 allele. Similarly, all of moderately differentiated OSCC patients and half of well-differentiated OSCC patients revealed null GSTM1 genotypes (Tanwar et al., 2015). Matthias et al also detected null GTST1 genotype in 19.1% of grade I or II diseases, while 46.2% of grade III OSCC patients (Jahnke et al., 1999). This pattern was similar to our results regarding the association of T1 null genotype with more aggressive behavior. Null genotype of T1 allele was also associated with significantly higher lymphatic metastasis in OSCC (Koch et al., 2010). Correlation of null genotypes of T1 and M1 polymorphisms with a prognosis of cancers of the oral cavity is to be more elucidated.

Mentioning limitations of our study, the results revealed no statistically significant difference regarding the distribution of the GST deletional variants between the case and control groups. This may be due to the relatively small sample size of our study delivering a low power to stablish such difference. However, obtaining large sample size in cancer studies is a concerning issue, and this is a limitation to many of studies in the field. It is recommended to explore the role of these polymorphisms with larger sample to clarify any statistically significant associations.

In conclusion, null genotypes of GTSM1 and GTST1 polymorphisms showed a higher prevalence in patients diagnosed with OSCC than healthy individuals. Individual null states may contribute the risk of OSCC, however, a combination of these seems to exert a pronounced effect. In addition, T1 null genotype may be a marker in a progression of OSCC. Larger studies are recommended to derive firm conclusions about the effects of these genotypes on OSCC pathogenesis.

**Figure 1:**
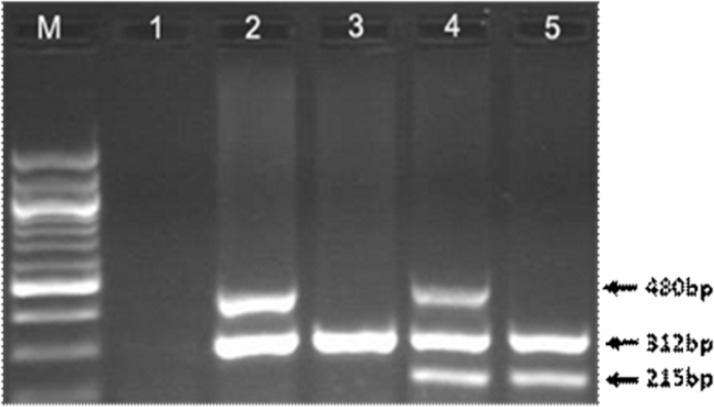
PCR Products Analyzed on 1.5% Agarose Gel. The presence of GSTT1 was determined by the presence of a band at 480 bp and GSTM1 was identified by the presence of a band at 215 bp. The 312 bp band was related to CYP1A1 gene as internal control. Lane 1 is negative control. Lane 2 represents a subject with only GSTT1 (480 bp) allele . Lane 3 is an individual with null alleles for both GSTT1 and GSTM1 genes showing only one band at 312 bp (internal control). Lane 4 is an individual with inheritance of both GSTT1 and GSTM1 alleles. Lane 5 is an individual with GSTT1 null and GSTM1 wild-type (215 bp) alleles. “M” denotes DNA marker

**Table 1:** Features of Oral Squamous Cell Carcinoma Patients

	Parameters	OSCC (N=50) N (%)
Tumor location	Mandibular gingiva	18 (36)
	Buccal mucosa	14 (28)
	Maxillary gingiva	7 (14)
	Tongue	5 (10)
	Labial mucosa	4 (8)
	Ventral surface of tongue	2 (4)
Tumor grade	Grade I	21 (42)
	Grade II	20 (40)
	Grade III	9 (18)

**Table 2:** Frequencies of GSTM1 and GSTT1 Genotypes in Oral Squamous Cell Carcinoma Patients and Healthy Individuals

		Patients N=50	Controls N=63	P value
		n (%)	n (%)	
GSTM1	+	21 (42)	36 (57.1)	0.11
	-	29 (58)	27 (42.9)	
GSTT1	+	38 (76)	53 (84.1)	0.28
	-	12 (24)	10 (15.9)	
GSTM1& GSTT1 combinations	TM (+), TT (+)	17 (34)	31 (49.2)	
	TM (+), TT (-)	5 (10)	5 (7.9)	0.39
	TM (-), TT (+)	21 (42)	22 (34.9)	
	TM (-), TT (-)	7 (14)	5 (7.9)	

**Table 3:** Distribution of GSTM1 and GSTT1 Genotypes in OSCC Patients with Different Histopathologic Grades

Genotypes		OSCC grade			P value
		Grade I	Grade II	Grade III	
		N=21	N=20	N=9	
		n (%)	n (%)	n (%)	
GSTM1	+	7 (33.3)	10 (50)	4 (44.4)	0.55
	-	14 (66.7)	10 (50)	5 (55.6)	
GSTT1	+	16 (76.2)	19 (95)	3 (33.3)	0.002*
	-	5 (23.8)	1 (5)	6 (66.7)	
GSTM1&	TM (+), TT (+)	6 (28.6)	10 (50)	1 (11.2)	
GSTT1 combinations	TM (+), TT (-)	1 (4.8)	0 (0)	4 (44.4)	0.01*
	TM (-), TT (+)	10 (47.6)	9 (45)	2 (22.2)	
	TM (-), TT (-)	4 (19)	1 (5)	2 (22.2)	

*, Fisher's Exact Test

**Table 4:** Statistical Analysis of GSTM1 and GSTT1 Genotypes in Oral Squamous Cell Carcinoma Patients

Genotypes		Odd ratio	95% Confidence interval	P value
GSTM1	+		Reference	
	-	1.8	0.9-3.9	0.11
GSTT1	+		Reference	
	-	1.7	0.7- 4.3	0.28
GSTM1& GSTT1	TM (+), TT (+)		Reference	
combination	TM (+), TT (-)	1.8	0.5- 7.2	0.39
	TM (-), TT (+)	1.7	0.7- 4	0.19
	TM (-), TT (-)	2.5	0.7-9.3	0.15

## Ethical approval

The study was approved by ethical committee of Zahedan University of Medical Sciences (IR.ZAUMS.REC.1395. 153). Written informed consent was acquired from the patients. 

## Conflict of interests

The authors declare that they have no conflict of interest.
